# Development and validation of risk prediction model for identifying 30-day frailty in older inpatients with undernutrition: A multicenter cohort study

**DOI:** 10.3389/fnut.2022.1061299

**Published:** 2023-01-11

**Authors:** Hongpeng Liu, Cheng Li, Jing Jiao, Xinjuan Wu, Minglei Zhu, Xianxiu Wen, Jingfen Jin, Hui Wang, Dongmei Lv, Shengxiu Zhao, Stephen Nicholas, Elizabeth Maitland, Dawei Zhu

**Affiliations:** ^1^School of Nursing, Peking University, Beijing, China; ^2^Department of Orthopaedic Surgery, Beijing Jishuitan Hospital, Fourth Clinical College of Peking University, Beijing, China; ^3^Center for Musculoskeletal Surgery (CMSC), Freie Universität Berlin, Humboldt-Universität zu Berlin, Berlin Institute of Health, Charitá-Universitätsmedizin Berlin, Berlin, Germany; ^4^Department of Nursing, Peking Union Medical College Hospital, Chinese Academy of Medical Sciences and Peking Union Medical College, Beijing, China; ^5^Chinese Nursing Association, Beijing, China; ^6^Department of Geriatrics, Peking Union Medical College Hospital, Chinese Academy of Medical Sciences and Peking Union Medical College, Beijing, China; ^7^Department of Nursing, Sichuan Provincial People's Hospital, Chengdu, China; ^8^Department of Nursing, The Second Affiliated Hospital Zhejiang University School of Medicine, Hangzhou, China; ^9^Department of Nursing, Tongji Hospital, Tongji Medical College, Huazhong University of Science and Technology, Wuhan, China; ^10^Department of Nursing, The Second Affiliated Hospital of Harbin Medical University, Harbin, China; ^11^Department of Nursing, Qinghai Provincial People's Hospital, Xining, China; ^12^Australian National Institute of Management and Commerce, Sydney, NSW, Australia; ^13^School of Economics and School of Management, Tianjin Normal University, Tianjin, China; ^14^Guangdong Institute for International Strategies, Guangdong University of Foreign Studies, Guangzhou, China; ^15^Newcastle Business School, University of Newcastle, University Drive, Newcastle, NSW, Australia; ^16^School of Management, University of Liverpool, Liverpool, United Kingdom; ^17^China Center for Health Development Studies, Peking University, Beijing, China

**Keywords:** models, risk assessment, older adults, undernutrition, biochemical parameters, frailty

## Abstract

**Objective:**

To develop and externally validate a frailty prediction model integrating physical factors, psychological variables and routine laboratory test parameters to predict the 30-day frailty risk in older adults with undernutrition.

**Methods:**

Based on an ongoing survey of geriatrics syndrome in elder adults across China (SGSE), this prognostic study identified the putative prognostic indicators for predicting the 30-day frailty risk of older adults with undernutrition. Using multivariable logistic regression analysis with backward elimination, the predictive model was subjected to internal (bootstrap) and external validation, and its calibration was evaluated by the calibration slope and its C statistic discriminative ability. The model derivation and model validation cohorts were collected between October 2018 and February 2019 from a prospective, large-scale cohort study of hospitalized older adults in tertiary hospitals in China. The modeling derivation cohort data (*n* = 2,194) were based on the SGSE data comprising southwest Sichuan Province, northern Beijing municipality, northwest Qinghai Province, northeast Heilongjiang Province, and eastern Zhejiang Province, with SGSE data from Hubei Province used to externally validate the model (validation cohort, *n* = 648).

**Results:**

The incidence of frailty in the older undernutrition derivation cohort was 13.54% and 13.43% in the validation cohort. The final model developed to estimate the individual predicted risk of 30-day frailty was presented as a regression formula: predicted risk of 30-day frailty = [1/(1+e^−*riskscore*^)], where riskscore = −0.106 + 0.034 × age + 0.796 × sex −0.361 × vision dysfunction + 0.373 × hearing dysfunction + 0.408 × urination dysfunction – 0.012 × ADL + 0.064 × depression – 0.139 × nutritional status – 0.007 × hemoglobin – 0.034 × serum albumin – 0.012 × (male: ADL). Area under the curve (AUC) of 0.71 in the derivation cohort, and discrimination of the model were similar in both cohorts, with a C statistic of nearly 0.7, with excellent calibration of observed and predicted risks.

**Conclusion:**

A new prediction model that quantifies the absolute risk of frailty of older patients suffering from undernutrition was developed and externally validated. Based on physical, psychological, and biological variables, the model provides an important assessment tool to provide different healthcare needs at different times for undernutrition frailty patients.

**Clinical trial registration:**

Chinese Clinical Trial Registry [ChiCTR1800017682].

## 1. Introduction

Population aging, the percentage of older adults in a country's population, is increasing globally and in China. It is estimated that China's population aged 65 and above will reach 365 million by 2050, accounting for a quarter (26.1%) of China's total population (1). Population aging is accompanied by an increased prevalence of undernutrition, or those suffering from a generally poor nutritional status (2–4). Several age-related pathophysiological and psychosocial factors, as well as protein and nutrient intake and drug use, shape dietary habits, leading to specific nutrition deficits (1, 5). Among the elderly admitted to hospital, undernutrition is a common and serious nutritional condition, which often deteriorates further during hospitalization (4, 6). The prevalence of hospital undernutrition is estimated to range from 30% to 50% of inpatients (4, 7, 8). Boulos et al. indicated that a significant association was found between undernutrition and frailty (*p* < 0.001), undernutrition was related to a nearly four-fold higher risk of frailty (6). Indeed, undernutrition's negative impact on health substantially increases the risk of frailty (9).

Frailty is the primary challenge for China's geriatric population (1, 10). Frailty is a biological syndrome characterized by deteriorating functions across a broad spectrum of physiological symptoms, accompanied by an increased vulnerability to stressors (11). Attracting increasing scientific interest in the past few decades, the investigation of frailty covers multiple organ systems, including the skeletal muscle, brain, immune system, cardiovascular system, respiratory system and endocrine system (12, 13). Importantly, the concept of frailty is multidimensional, with physical, nutritional, and psychosocial factors contributing to frailty (10). Since frailty is preventable and treatable up to the point of no return, which occurs when frailty indicates a pre-death phase, assessments to detect frailty are crucial (14).

Previous research has developed measurement tools for identifying frailty (11, 15–18), including the physical phenotype model of Fried et al. (11), the FRAIL scale (18), the deficit accumulation models of Mitnitski et al. (15) and Rockwood et al. (16) and the mixed physical and psychosocial models, represented by the Tilburg Frailty Indicator (TFI) (17) and Edmonton Frailty Scale (EFS) (19). The biological and theoretical bases differ among these assessment tools, and most of these tools were developed by traditional modeling methods (20). Despite previous reports suggesting that reliable frailty models should be underpinned by biological principles of causality, few frailty prediction tools include routine laboratory test results (12). Further, the frailty research focus is moving toward prediction tools for specific settings and populations (10, 21–23). While both the integral conceptual frailty model (24) and the phenotype model (11) include nutritional status as a factor to explain frailty, neither model provides a specific tool for undernutrition populations. Additionally, none of the current prediction tools were developed on data from the Asia-Pacific region, which has the world's greatest senior population together with a diverse population in terms of socioeconomics, access to healthcare facilities, and ethnicity, especially among the Chinese (25, 26).

We address these lacunae by hypothesizing that the patient's characteristics, physical and psychological factors and the results of routine laboratory tests can predict patients' 30-day frailty risk. Specifically, we develop and externally validate a frailty prediction model integrating the patient characteristics, physical and psychological factors and the results of routine laboratory tests to predict the frailty risk in older adult inpatients with undernutrition. Our model directly informs patient health care.

## 2. Methods

### 2.1. Data sources and patients

From a large-scale geriatric syndrome cohort survey of hospitalized elderly adults across China (SGSE, Chinese Clinical Trial Registry Number: ChiCTR1800017682) (4, 20), all eligible older inpatients who lived in the internal wards and surgical wards of these selected hospitals in the study period (between October 2018 and February 2019) were considered as the study subjects, as long as they met the inclusion criteria. All included patients were 65 years old or older and gave their informed consent to participate in the study. Providing national coverage, the tertiary hospitals were located in eastern Zhejiang Province, south-central Hubei Province, southwest Sichuan Province, northeast Heilongjiang Province, northern Beijing municipality and northwest Qinghai Province.

**Figure 1:**
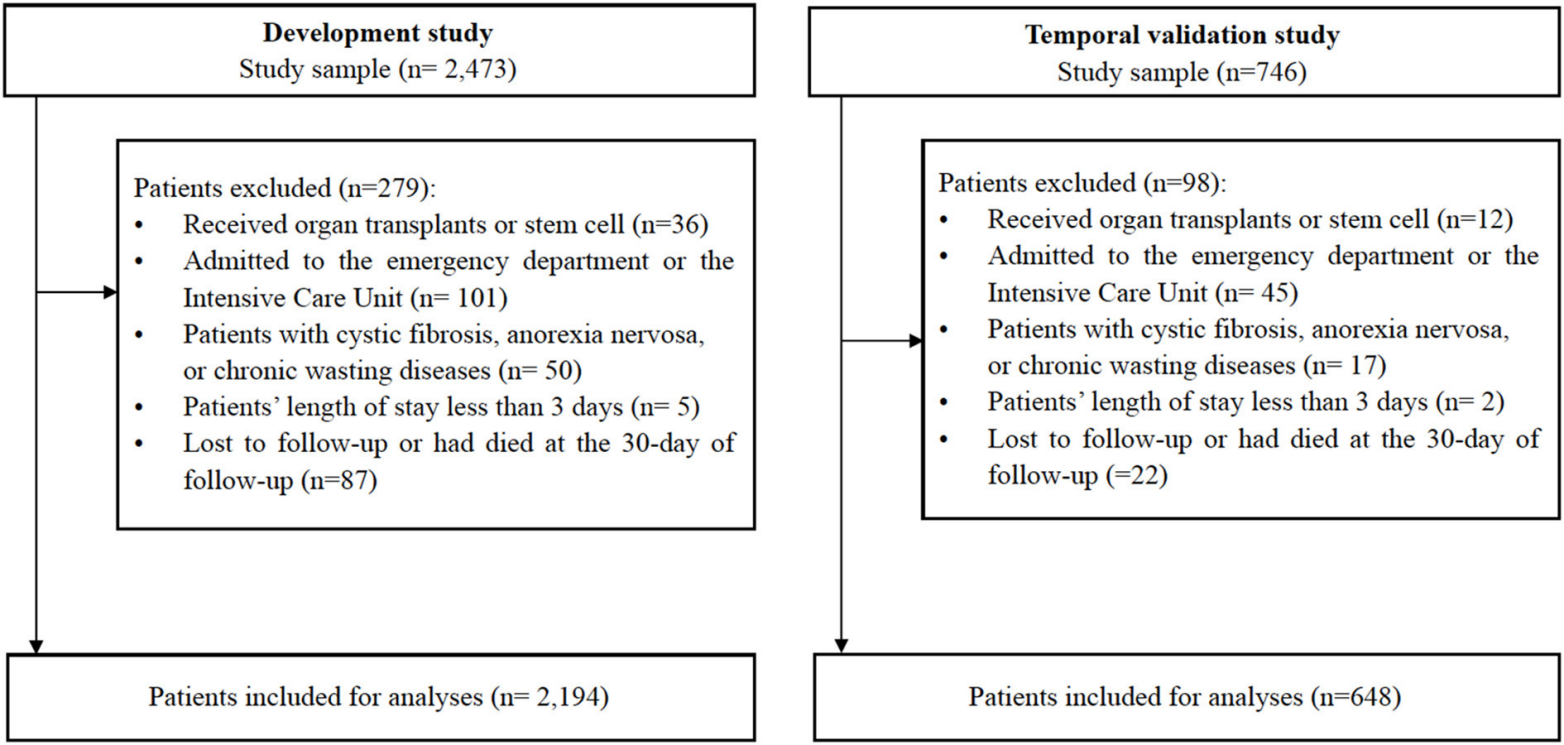
Inclusion/exclusion cascade of patients in the dataset (*n* = 2,842).

Our sample comprised 2,842 inpatients aged 65 years and older, with no frailty according to the FRAIL scale (scores range from 0 to 2) and with undernutrition (scores range from 0 to 11) according to the Mini-Nutritional Assessment-Short Form (MNA-SF). MNA-SF is a six-item instrument with scores ranging from 0 to 14 points. For the purpose of our study, MNA-SF scores range from 0 to 11 referring to undernutrition, and undernutrition includes malnutrition risk and malnourished. Therefore, the nutritional status means at risk of malnutrition (scores ranging from 8 to 11 points) and malnourished (scores ranging from 0 to 7 points). To ensure data quality, the surveys are managed by trained nurses using a structured Case Report Form (CRF), the nurses received training and test before they apply the assessment to the patients. All CRF results were reviewed by the research team. The outcomes were assessed by the professional nurses who have cared for these older inpatients using the FRAIL scale at the 30-day follow-up. As shown in Figure 1, we excluded patients who received organ transplants or stem cell transplantation; patients who admitted to the emergency department or intensive care unit (ICU); patients with persistently unconsciousness, acute pancreatitis, acute infectious diseases, acute liver failure; patients with cystic fibrosis, anorexia nervosa, severe chronic gastrointestinal diseases, or chronic wasting diseases at enrollment; and patients who were lost to follow-up or had passed away within 30-days of follow-up. For the predictive modeling, 2,194 observations were used from the SGSE data set and 648 inpatients from the Hubei Province were used to externally validate the model.

### 2.2. Defining frailty

The International Working Group on Nutrition, Health and Aging proposed the FRAIL scale as a clinical frailty screening tool in 2008. It has 5 straightforward self-reported questions on topics including fatigue, resistance, ambulation, illness, and loss of weight. The frail (scores range from 3 to 5), pre-frail (scores range from 1 to 2), and robust categories on the FRAIL scale run from 0 (best) to 5 (worst) (0) (18). The FRAIL scale has been validated for use in older Chinese inpatients (27). In the current study, the outcome variable was frailty (FRAIL 3 to 5) at the 30-day follow-up.

### 2.3. Ethics

This study was approved by the Ethics Review Board in Peking Union Medical College Hospital (S-K540). Written informed consent was provided by all participants enrolled in the study. When a patient was unable to answer the questions themselves, their spouse or other legal guardians were interviewed.

### 2.4. Candidate predictors, missing data, and power calculations

Based on a literature review, 29 patient characteristics were selected from the SGSE database for the 2,194 inpatient derivation cohort. We also added predictors identified in previous studies and in clinical practice as important frailty risk factors (25, 28) comprising age, sex, marital status, vision, hearing, sleeping, urinary function, defecation function, handgrip strength, activities of daily living (ADL), instrument activities of daily living (IADL), cognitive function, nutritional status, depression, red blood cell (RBC), serum albumin, hemoglobin, neutrophils, white blood cell (WBC), number of medications, serum potassium, serum sodium, blood urea nitrogen, creatinine, C-reactive protein, BMI, education level, smoking, and alcohol consumption.

The Barthel Index (BI), a 10-item questionnaire measuring disability in terms of a person's level of functional independence in personal ADL, was used to evaluate ADL.

Each of the ten ADL in BI is rated as 0, 5, or 10, with a possible overall score of 100.

A higher grade indicates a better ability to carry out daily activities (20). The depression assessment scale was developed on the basis of the Geriatric Depression Scale 15 (GDS15) (29), for it, patients were asked if they had ever experienced sadness, blueness, or depression for 2 weeks or longer in a row in previous year. They were asked 15 questions on their satisfaction with life, whether they had lost interest in activities, were exhausted or low energy, had more trouble concentrating than normal, had been thinking a lot about death and felt useless, etc. Each response received a score of one or zero. The aggregate of the answers to these 15 questions about depression made up the final GDS score (range 0–15). The patient was deemed to be depressed if their overall GDS score was >5, with a higher score denoting more severe depression.

Our derivation cohort had some missing information on BMI (1.37% of the sample), handgrip strength (3.42% of the sample), depression (4.97% of the sample), cognitive function (5.06% of the sample), and nutritional status (0.87% of the sample). Following Sultan et al. (30), multiple imputations were used to replace missing values by using a chained equation approach based on all candidate predictors (31). Five imputed datasets for missing variables were created, which were then combined across all datasets by using Rubin's rule to obtain final model estimates (30). When a routine laboratory test parameter could not be obtained, there was no reason to believe the missing variable had an abnormal value, thus we used the mean normal value to impute the missing variable's value (31). To determine the normal value, we first identified all participants who had a normal value, then computed the mean value for this group of participants and utilized it for imputation. For the validation data, we used the method described above to impute missing data for BMI (4.94% of the sample), handgrip strength (8.80% of the sample), depression (7.72% of the sample), cognitive function (5.71% of the sample), and nutritional status (3.7% of the sample).

Based on an estimated 297 frailty events during the 30-day follow-up period and 29 candidate parameters in the derivation cohort, the effective sample size of the 11 frailty events per predictor was above the minimum requirements (32).

### 2.5. Model development and validation

The occurrence of frailty at the 30-day follow-up was defined as the binary outcome measure. We used restricted cubic splines to model potential non-linear associations between frailty and the continuous parameters. We included interaction terms to test potential sex differences in the predictors. To derive the prediction model of frailty, we included all predictors in a logistic regression model with backward elimination to evaluate the association of each potential risk factor with frailty outcomes. The absence of multicollinearity and plausible interactions among variables were evaluated to ensure the robustness of the logistic regression model. We used the estimated β coefficients multiplied by the corresponding parameters, together with the average intercept across patient clusters, to form the risk model for predicting the log odds of frailty (30).

Performance of the frailty model was evaluated using the C statistic and calibration slope (where 1.00 is the ideal value) (30). The C statistic, where 0.50 indicates no discrimination and 1.00 indicates perfect discrimination, measures the likelihood that, for every randomly chosen pair of patients with and without frailty, the frailty patient had a greater predicted risk (30). By bootstrapping 100 samples of the derivation data, we did internal validation to correct measures of predictive performance for optimism (over-fitting) (31). The model was used to test model performance (calibration slope and C statistic) and optimism on the original dataset (difference in test performance and apparent performance). The shrinkage coefficient, which was equal to the average calibration slope from each of the bootstrap samples, was then used to estimate the overall optimism across all models. The original β coefficients were multiplied by the uniform shrinkage factor in the final model in order to account for over-fitting during the development process. To maintain overall calibration, the intercept based on the shrunken coefficients was re-estimated at this step, producing a final model (30).

Our risk prediction model was applied to each patient in the external validation cohort on the basis of the presence of risk factors (31). The C statistic assessed the discrimination performance of the final model and we evaluated calibration by charting agreement between predicted and observed risks across one-tenths of the predicted risk. For the external validation in the Hubei Province data, our study re-calibrated the intercept on the basis of the incidence of frailty and mean centering of all predictors (30). Details on the parameters in the predicted absolute risk of frailty model are provided in Supplementary Table 1.

Next, we carried out a decision curve analysis to assess the utility of the model for decision-making. Decision curve analysis is a technique to assess the clinical net benefit of prediction models, where the weights assigned to true positives (benefits) and false positives (harms) are derived from the threshold probability of the outcome (33). This is then used to determine the model's overall net benefit for a variety of threshold probabilities. The clearest way to evaluate a decision curve is to say that it has the highest clinical value if it has the most net benefit at a given threshold (31).

R version 4.1.1 was utilized for all of our statistical calculations. The Transparent Reporting of a multivariate prediction model for Individual Prediction or Diagnosis (TRIPOD) guidelines were followed when conducting and reporting this study. All reported *P* values are two-sided and statistical significance was set at 0.05.

## 3. Results

### 3.1 Study participants

As shown in Figure 1, we analyzed SGSE derivation cohort data on 2,194 older inpatients admitted to tertiary hospitals with a complete 4 weeks of telephone follow-up. Our validated Hubei cohort had information on 648 older inpatients, meeting the same conditions as the derivation cohort. Table 1 shows the characteristics of the study participants. Broadly, participants in both cohorts had similar prevalence of frailty, male sex, ADL, and nutritional status. Compared with the derivation cohort, participants in the Hubei validation cohort were less likely to have vision and hearing dysfunction (*P* < 0.001), and more likely to have urination dysfunction (*P* < 0.001).

**Table 1:** Participants' characteristics in cohort studies.

**Variable**	**Derivation cohort (*n* = 2,194)**	**Validation cohort (*n* = 648)**
Frailty, *N* (%)	297 (13.54)	87 (13.43)
Male, *N* (%)	1252 (57.07)	381 (58.80)
Vision dysfunction, *N* (%)	480 (21.88)	98 (15.12)^***^
Hearing dysfunction, *N* (%)	439 (20.01)	85 (13.12)^***^
Urination dysfunction, *N* (%)	251 (11.44)	116 (17.90)^***^
Age (years), mean (SD)	72.12 (5.78)	71.18 (5.44)
ADL, mean (SD)	90.75 (17.91)	91.14 (16.96)
Depression, mean (SD)	3.14 (2.88)	4.50 (3.32)
MNA-SF scores, mean (SD)	8.43 (2.07)	8.48 (1.96)
Hemoglobin (g/L), mean (SD)	126.59 (21.95)	120.04 (20.66)
Serum albumin (g/L), mean (SD)	37.95 (5.67)	39.30 (5.32)

Values are expressed in absolute (relative) frequencies unless stated otherwise; MNA-SF scores refers to MNA-SF scores ranging from 0 to 11 points. ^***^P < 0.001.

ADL, activities of daily living.

### 3.2. Model development, performance measure, and validation

**Figure 2:**
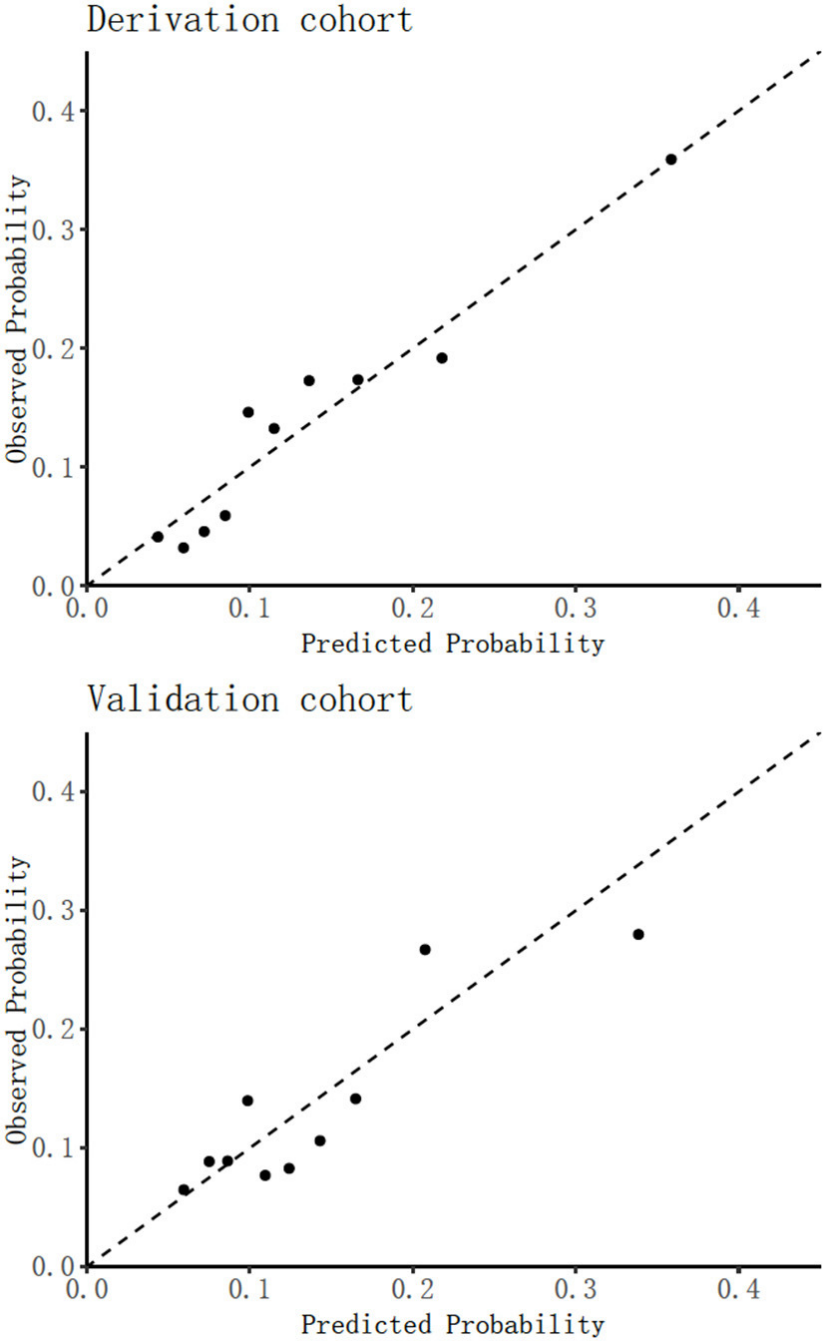
Assessing calibration in derivation cohort **(top)** and validation cohort **(bottom)**.

In the derivation cohort, Table 1 shows that 297 frailty events occurred during the first 4 weeks of follow-up with an absolute rate of 13.54%. Table 2 indicates that among the 29 potential parameters, 11 were statistically significantly associated with frailty in the multivariable model. The final model developed to estimate the individual predicted risk of 30-day frailty was shown as a regression formula: predicted risk of 30-day frailty = [1/(1+e^−*riskscore*^)], where riskscore = −0.106 + 0.034 × age + 0.796 × sex −0.361 × vision dysfunction + 0.373 × hearing dysfunction + 0.408 × urination dysfunction – 0.012 × ADL + 0.064 × depression – 0.139 × nutritional status – 0.007 × hemoglobin – 0.034 × serum albumin – 0.012 × (male: ADL). Table 3 reports the apparent and internal validation performance statistics of the model. The final risk prediction model could distinguish between the elderly who were frail and those who weren't after accounting for optimism, with a C statistic of 0.694 (95% of confidence interval 0.659–0.730). Figure 2 (top) illustrates the agreement between the observed and predicted proportion of events, displaying excellent apparent calibration, with a uniform shrinkage factor of 0.806 used to adjust predictor coefficients in the final prediction model for optimism in Table 3.

**Table 2:** Final multivariable analysis for frailty risk within 30 days of admission in derivation cohort.

**Term**	**β coefficients**	**SE**	***P***	**OR**	**95% CI**	
(Intercept)	−0.106	1.087	0.922	0.899	0.107	7.584
Age (years), mean (SD)	0.034	0.011	0.002	1.034	1.012	1.057
Male, *N* (%)	0.796	0.519	0.126	2.217	0.800	6.139
Vision dysfunction, *N* (%)	−0.361	0.184	0.050	0.697	0.486	1.000
Hearing dysfunction, *N* (%)	0.373	0.178	0.036	1.452	1.024	2.059
Urination dysfunction, *N* (%)	0.408	0.188	0.030	1.503	1.041	2.172
ADL, mean (SD)	−0.012	0.004	0.003	0.988	0.980	0.996
Depression, mean (SD)	0.064	0.023	0.006	1.066	1.019	1.116
Nutritional status, mean (SD)	−0.139	0.030	< 0.001	0.870	0.821	0.922
Hemoglobin (g/L), mean (SD)	−0.007	0.003	0.030	0.993	0.988	0.999
Serum albumin (g/L), mean (SD)	−0.034	0.011	0.003	0.966	0.945	0.988
Male: ADL	−0.012	0.006	0.038	0.988	0.977	0.999

ADL, activities of daily living; OR, odds ratio; CI, confidence interval.

**Table 3:** Model diagnostics (with 95% CI).

	Apparent performance^*^	**Test performance^†^**	**Average optimism^‡^**	**Optimism corrected^§^**	**External validation**
C statistic	0.733 (0.698, 0.769)	0.694 (0.678, 0.707)	0.039	0.694 (0.659, 0.730)	0.645 (0.575, 0.710)
Calibration slope	−0.32 (−0.572, −0.137)	0.806 (0.695, 0.937)	0.194	0.806 (0.695, 0.937)	0.879

CI, confidence interval.

* Refers to performance estimated directly from dataset that was used to develop prediction model.

† Determined by developing model in each bootstrap sample (100 samples with replacement), calculating performance (bootstrap performance), and applying bootstrap model in original sample.

‡ Average difference between model performance in bootstrap data and test performance in original dataset.

§ Subtracting average optimism from apparent performance.

### 3.3. External validation

In our Hubei Province validation cohort in Table 1, 648 older adults had frailty with an absolute rate of 13.43%. Applying our final frailty risk prediction model to the Hubei data, after recalibration of the intercept, gave a C statistic of 0.645 (0.575–0.710) indicating excellent calibration [43], which is plotted in Figure 2 (bottom), with the calibration slope of 0.879, as shown in Table 3.

### 3.4. The utility of the frailty prediction model for decision making

**Figure 3:**
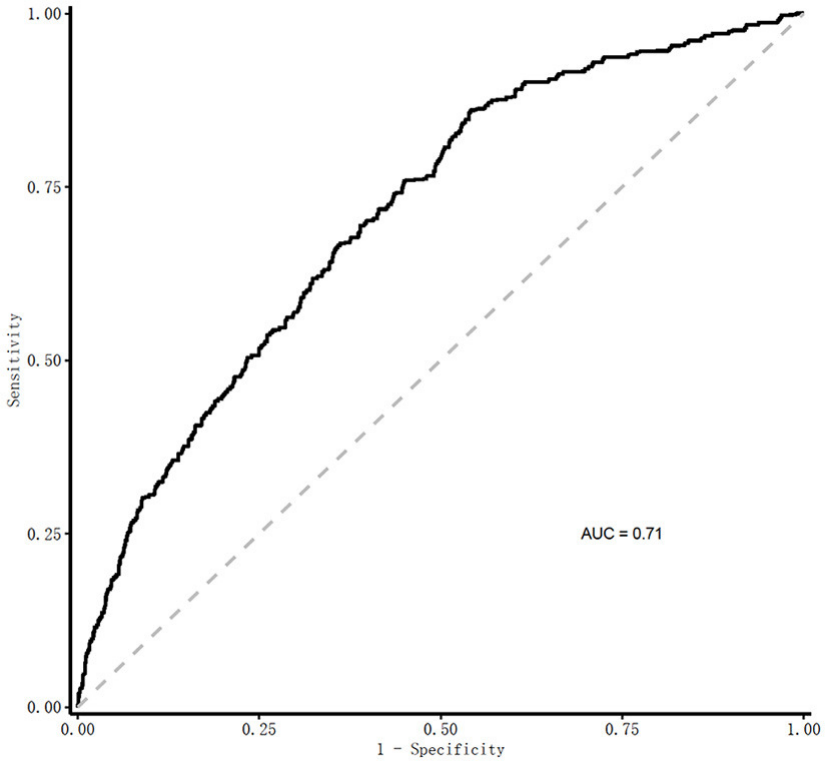
AUC for the detection of frailty by the frailty prediction model. Predicted risk of 30-day frailty = [1/(1+e^−*riskscore*^)], where riskscore = −0.106+0.034 × age+0.796 × sex −0.361 × vision dysfunction+0.373 × hearing dysfunction+0.408 × urination dysfunction −0.012 × ADL+0.064 × depression −0.139 × nutritional status −0.007 × hemoglobin −0.034 × serum albumin – 0.012 × (male: ADL). ROC, receiver operating characteristic curve; AUC, Area Under Curve.

**Figure 4:**
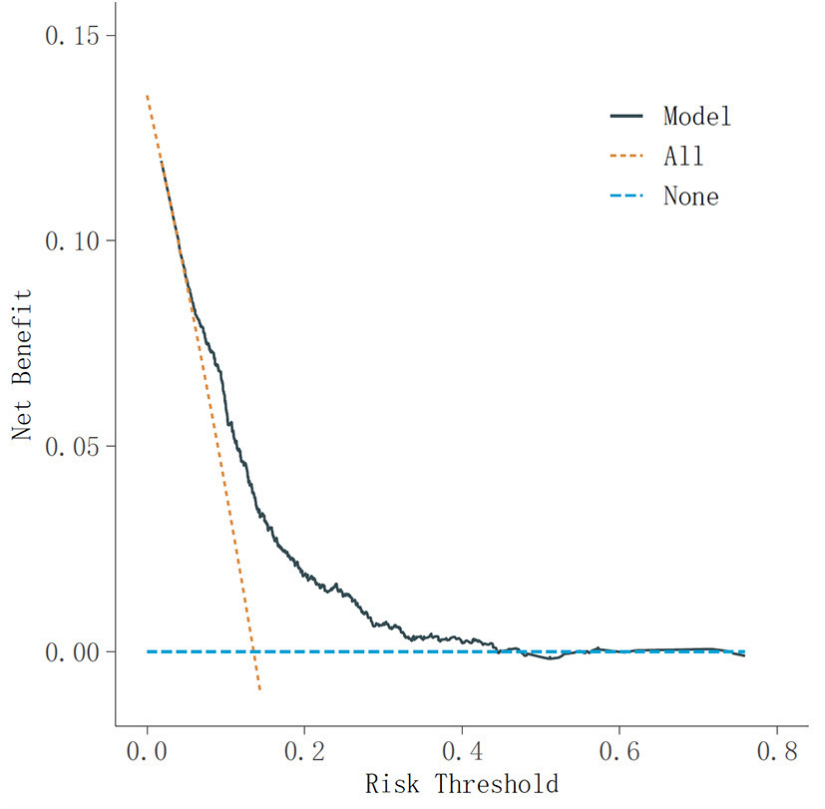
Decision curve analysis of the prediction model for frailty detection. DCA, decision curve analysis.

In the receiver operating characteristic (ROC) curve analyses in Figure 3, the area under curve (AUC) of the frailty risk prediction model was 0.71. Figure 4 shows the decision curve analysis of the prediction model for frailty detection. In Figure 4, the yellow line farthest left represents the “screening all” strategy and the horizontal blue line indicates the “screening none” strategy. For predicted probability thresholds between 5% and 45%, the new frailty prediction model showed a positive net benefit.

## 4. Discussion

Our study is among the first to develop a new risk prediction model for identifying frailty among hospitalized older adults with undernutrition. The current frailty risk prediction model was developed according to the FRAIL scale, which is based on elements of Fried phenotype and a method of counting an individual's illnesses and disabilities (11, 18, 34). We developed our model by examining the psychological and initial biochemical data in representative samples of Chinese elderly inpatients, which was externally validated using a Hubei Province cohort. By applying a well-established theoretical framework of frailty, and followed international guidelines, our prediction model had excellent calibration and useful discrimination, with a C statistic of nearly 0.7 in both the derivation (AUC 0.71) and validation data.

Implementation of the frailty risk prediction model among older inpatients offers a major advance in the detection of frailty and the care of older patients with frailty. For instance, from the perspective of the predictive values that were defined by the model, age, vision, hearing, and urination dysfunction, and depression were the strongest predictors of frailty risk in the final multivariable model. This suggests that clinical medical staff should pay attention to the above problems after the elderly is hospitalized. Once the above problems are found, goal-orientated care can be implemented promptly.

From the perspective of healthcare policies, frailty is the primary challenge for the geriatric population (10). Health policymakers can refer to the research results of this paper to propose risk prevention and management strategies for frail older adults. Meanwhile, to strengthen propaganda and raise the public's awareness of frailty.

### 4.1. Strengths of the study

Our frailty risk prediction model has several advantages. The model is based on absolute risks determined and validated in an independent Asian population. It is developed using widely accessible clinical and demographic data, making it simple to apply in clinical practice and easily adaptable to additional external validation in other nations with readily accessible routine data for such a purpose. Besides, routine identification of frailty in clinical settings, the frailty prediction model promises to improve both hospital care and secondary care and specialist services, such as palliative care.

Previous frailty models lacked reliability in specific settings, and it is unclear which frailty tool should be applied in which setting (21, 23). To compound these issues, other frailty tools do not identify the same individuals or predict the same adverse outcomes (35). Additionally, the frailty field is currently moving toward specific tools for specific settings and populations (10), such as the Hospital Frailty Risk Score (HFRS) and the electronic Frailty Index (eFI) (21–23). Since nutritional status often deteriorates further during hospitalization (4, 6), undernutrition negatively impacts and substantially increases the risk of frailty. Establishing specific tools for different groups of patients is overdue, which allows individually tailored nutritional interventions. Previous studies indicated that psychosocial factors such as depression should be included in the frailty assessment process (36). While Gobbens et al.'s (17) TFI includes psychometric properties, TFI is a self-report assessment tool for measuring frailty in older adults. Addressing these issues, we developed and validated a specific tool among undernutrition older Chinese inpatients, which directly informs inpatient health care. Based on real-world data, which includes depression, our model is an examiner-rating tool that can be used by nurses, physicians, or even family members who have long-term care experience.

Translating from frailty research to clinical practice is one of the critical challenges for health care and treatment (25). For healthcare professionals, the clinometric aspects of assessment tools are fundamental for deriving precise biomarkers of frailty that will facilitate the development of more precise treatment strategies (35). Multidimensional modeling of a panel of complementary biomarkers, including biological parameters, are needed (37). Currently, there are no endorsed biomarkers of frailty available to clinicians, regulatory authorities, and academics (37, 38). Considering the changes of objective biological indicators in the blood are more scientific than those of subjective evaluation, our model encompasses initial biochemical data, such as serum albumin and hemoglobin, which promotes the identification of those who are frail and allows better tracking of frailty over time. Additionally, underpinned by biological principles of causality, with the changes of objective biological indicators in blood, our model is easier monitoring of whether interventions are effective (12).

### 4.2. Limitations of the study

There are limitations to the current study that need discussion. Further predictive validity of the model needs to be assessed in a longitudinal study, with further validation of the model and emphasis on how frailty and its domains affect adverse clinical outcomes in the long term. Second, further study is needed to confirm the causal relationship by investigating the longitudinal change in each parameter over time. While not yet validated for settings other than tertiary hospitals, the model promises potential applications in non-tertiary hospitals and primary care settings. We recommend the examination of the model's validity in non-tertiary hospital settings. Third, data limitations mean that disease severity was not included in the model, which is a limitation shared with most of the existing frailty assessment tools. Take kidney disease as an example. The influence of mild kidney disease and moderate to severe kidney disease on frailty is different. However, the current model encompasses objective biological indicators, including serum albumin and hemoglobin. Serum albumin can reflect the severity of disease, this has overcome the limitations of previous research to some extent.

## 5. Conclusions

To our knowledge, this is the first study to develop and validate a frailty prediction model integrating physical, psychological, and biological parameters to predict the 30-day frailty risk in older adults with undernutrition in China. Our study provides an important tool to address the healthcare needs of older undernutrition frailty patients; to further advance evidence-based treatment options; and to identify cost-effective care delivery strategies to prevent robust health declining to frailty, functional disability and premature death.

## Data availability statement

The raw data supporting the conclusions of this article will be made available by the authors, without undue reservation.

## Ethics statement

The studies involving human participants were reviewed and approved by Peking Union Medical College Hospital (S-K540). The patients/participants provided their written informed consent to participate in this study.

## Author contributions

Study concept and design: HL, DZ, XWu, and JJia. Analysis and interpretation of data: DZ. Editing of the manuscript and drafting of tables: HL, DZ, CL, SN, and EM. Critical review of the manuscript for important intellectual content: HL, DZ, CL, SN, EM, and XWu. Patient recruitment, data collection, and manuscript editing: JJia, MZ, XWe, JJin, HW, DL, and SZ. All authors critically reviewed and approved the manuscript before it was submitted.
