# Supplementary material for: Development and validation of risk prediction model for identifying 30-day frailty in older inpatients with undernutrition: A multicenter cohort study
Source: Front Nutr. 2023 Jan 11;9:1061299. doi: 10.3389/fnut.2022.1061299 (PMC9874615; doi:10.3389/fnut.2022.1061299)
Supplement: Supplementary file 1 [file Table_1.docx]

**Supplementary Table 1. Candidate predictors and parameters in the final multivariable of the model.**

|  | Candidate predictors | Parameters in the final multivariable of the model | |  |
| --- | --- | --- | --- | --- |
| Demographic characteristics  ***x*_a1_** | Age | Age | Continuous, Years | |
| ***x*_a2_** | Sex | Sex | Categorical, 1 = Male, 0 = Female | |
| ***x*_a3_**  ***x*_a4_**  ***x*_a5_**  ***x*_a6_** | Marital status  Education level  Smoking  Alcohol consumption |  | Categorical, 1= Single, 0 = Married  Categorical, 0 = Illiterate, 1 = Primary school, 2 = Junior high school, 3 = High school and above  Categorical, 0 = Non-smoker, 1= Current smoker, 2= Former smoker  Categorical, 0 = Non-drinker, 1 = Current drinker, 2 = Former drinker | |
| Clinical examinations |  |  |  | |
| ***x*_b1_** | Vision dysfunction | Vision dysfunction | Categorical, 1 = Yes, 0 = No | |
| ***x*_b2_**  ***x*_b3_** | Hearing dysfunction  Urination dysfunction | Hearing dysfunction  Urination dysfunction | Categorical, 1 = Yes, 0 = No  Categorical, 1 = Yes, 0 = No | |
| ***x*_b4_**  ***x*_b5_**  ***x*_b6_** | ADL  Depression  Nutritional status | ADL  Depression  Nutritional status | Continuous, measured using the BI  Continuous, measured using the GDS15  Continuous, measured using the MNA-SF | |
| ***x*_b7_** | Sleeping dysfunction |  | Categorical, 1 = Yes, 0 = No | |
| ***x*_b8_** | Defecation dysfunction |  | Categorical, 1 = Yes, 0 = No | |
| ***x*_b9_** | Handgrip strength (kg) |  | Continuous | |
| ***x*_b10_** | IADL |  | Continuous, measured using the IADL | |
| ***x*_b11_** | Cognitive function |  | Continuous, measured using the Mini-Cog | |
| ***x*_b12_** | BMI (kg/m^2^) |  | Categorical, 0 = BMI <19, 1 = 19≤BMI<21, 2 = 21≤BMI<23, 3 = BMI≥23 | |
| ***x*_b13_** | Number of medications |  | Continuous | |
| Laboratory test results  ***x_c_*_1_**  ***x_c_*_2_**  ***x_c3_***  ***x_c4_*** | Serum albumin (g/L)  Hemoglobin (g/L)  Neutrophils (×10^9^/L)  RBC (×10^12^/L) | Serum albumin (g/L)  Hemoglobin (g/L) | Continuous  Continuous  Continuous  Continuous | |
| ***x_c5_***  ***x_c6_*** | WBC (×10^9^/L)  Serum potassium (mmol/L) |  | Continuous  Continuous | |
| ***x_c7_***  ***x_c8_***  ***x_c9_***  ***x_c10_*** | Serum sodium (mmol/L)  Blood urea nitrogen (mmol/L)  Creatinine (μmol/L)  C-reactive protein (mg/L) |  | Continuous  Continuous  Continuous  Continuous | |

Abbreviations, ADL, Activities of daily living; BI, Barthel Index; GDS15, Geriatric Depression Scale 15; MNA-SF, Mini Nutritional Assessment Short-Form; IADL, The Instrumental Activities of Daily Living Scale; RBC, red blood cell; WBC, white blood cell; BMI, body mass index.

Notes: According to a literature review and previous clinical practice, 29 candidate predictors were selected from the SGSE database for the 2194 inpatient derivation cohort.

.
